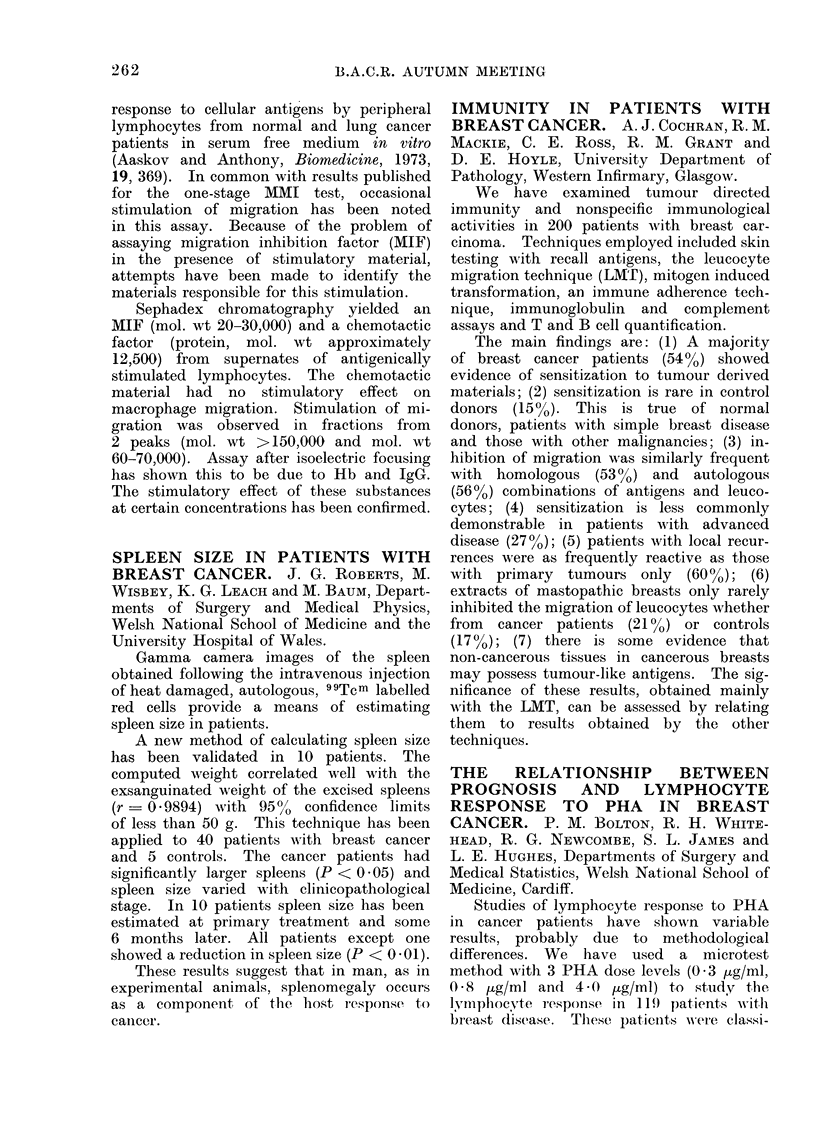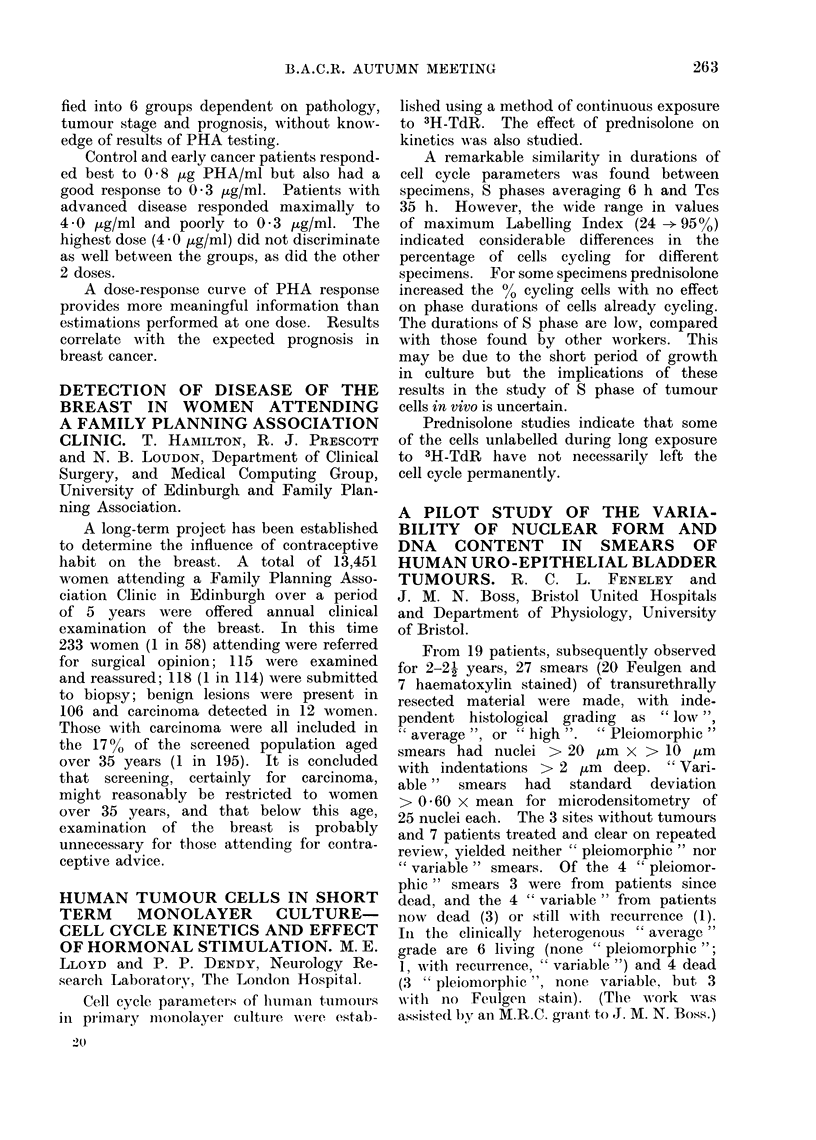# Proceedings: The relationship between prognosis and lymphocyte response to PHA in breast cancer.

**DOI:** 10.1038/bjc.1975.45

**Published:** 1975-02

**Authors:** P. M. Bolton, R. H. Whitehead, R. G. Newcombe, S. L. James, L. E. Hughes


**THE RELATIONSHIP BETWEEN PROGNOSIS AND LYMPHOCYTE RESPONSE TO PHA IN BREAST CANCER.** P. M. Bolton, R. H. Whitehead, R. G. Newcombe, S. L. James and L. E. Hughes, Departments of Surgery and Medical Statistics, Welsh National School of Medicine, Cardiff.

Studies of lymphocyte response to PHA in cancer patients have shown variable results, probably due to methodological differences. We have used a microtest method with 3 PHA dose levels ($0{\cdot}3$ $\mu$g/ml, $0{\cdot}8$ $\mu$g/ml and $4{\cdot}0$ $\mu$g/ml) to study the lymphocyte response in 119 patients with breast disease. These patients were classified into 6 groups dependent on pathology, tumour stage and prognosis, without knowedge of results of PHA testing.

Control and early cancer patients responded best to 0·8 μg PHA/ml but also had a good response to 0·3 μg/ml. Patients with advanced disease responded maximally to 4·0 μg/ml and poorly to 0·3 μg/ml. The highest dose (4·0 μg/ml) did not discriminate as well between the groups, as did the other 2 doses.

A dose-response curve of PHA response provides more meaningful information than estimations performed at one dose. Results correlate with the expected prognosis in breast cancer.